# Supplementary material for: Identification of photoperiod-induced specific miRNAs in the adrenal glands of Sunite sheep (Ovis aries)
Source: Front Vet Sci. 2022 Jul 22;9:888207. doi: 10.3389/fvets.2022.888207 (PMC9354845; doi:10.3389/fvets.2022.888207)
Supplement: Supplementary file 1 [file Data_Sheet_1.ZIP › Supplementary file/miRDeep2 file.zip!/miRDeep2 file/Know_Structure/LP42A1/oar-mir-411b.pdf]

miRBase precursor : oar-mir-411b
Total read count : 1517
oar-miR-411b-5p read count : 1505
oar-miR-411b-3p read count : 12
remaining reads : 0

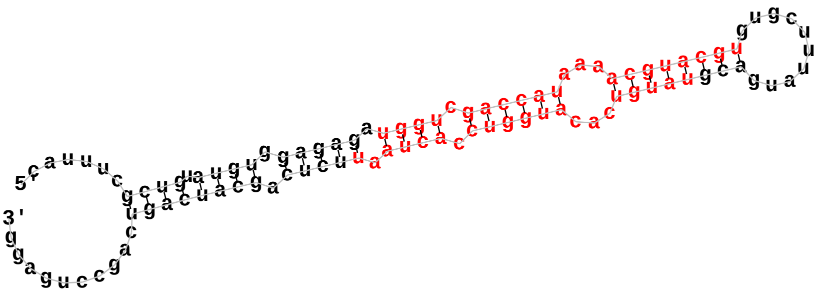

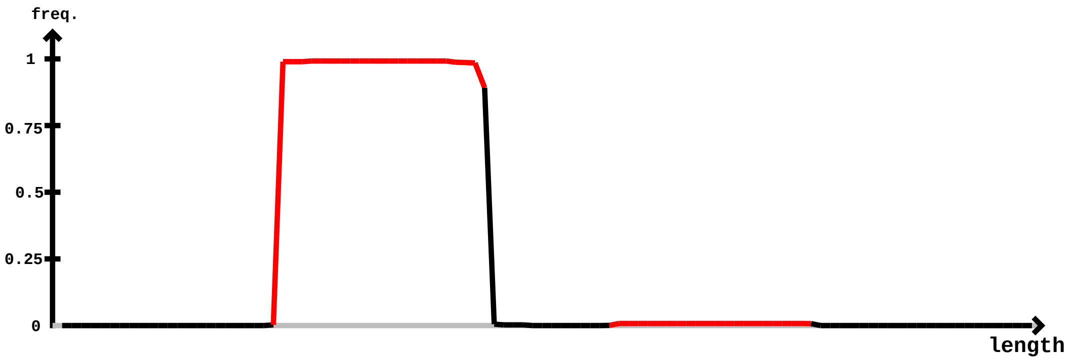


```
                                                           oar-miR-411b-3p
                        oar-miR-411b-5p
5'- cauuucgcuguauguggagagauggucgaccauaaaacguacgugugcuuuaugacguaugucacaugguccacuaauucucagcaucagucagccugagg   -3'    exp
    ......((((.((((.(((((.((((.((((((...((((((((..........))))))))...)))))).)))).))))).))))))))..........   reads     mm     sample
    .....................auggucgaccauaaaacguac...........................................................   1         0      seq
    .....................auggucgaccauaaaacguacg..........................................................   3         0      seq
    ......................uggucgaccauaaaacgu.............................................................   7         0      seq
    ......................uggucgaccauaaaacgua............................................................   2         0      seq
    ......................uggucgaccauaaaacguac...........................................................   1         0      seq
    ......................uggucgaccauaaaacguacg..........................................................   138       0      seq
    ......................uggucgaccauaaaacguacgu.........................................................   1345      0      seq
    ......................uggucgaccauaaaacguacgug........................................................   4         0      seq
    .........................ucgaccauaaaacguacgugugc.....................................................   4         0      seq
    ........................................................guaugucacaugguccacuaau.......................   1         0      seq
    .........................................................uaugucacaugguccacuaa........................   1         0      seq
    .........................................................uaugucacaugguccacuaau.......................   10        0      seq
```
